# Deep learning for multi-class semantic segmentation enables colorectal cancer detection and classification in digital pathology images

**DOI:** 10.1038/s41598-023-35491-z

**Published:** 2023-05-24

**Authors:** John-Melle Bokhorst, Iris D. Nagtegaal, Filippo Fraggetta, Simona Vatrano, Wilma Mesker, Michael Vieth, Jeroen van der Laak, Francesco Ciompi

**Affiliations:** 1 Department of pathology, Radboud University Medical Center, Nijmegen, The Netherlands; 2 Pathology Unit Gravina Hospital, Gravina Hospital, Caltagirone, Italy; 3 Leids Universitair Medisch Centrum, Leiden, The Netherlands; 4 Klinikum Bayreuth, Friedrich-Alexander-University Erlangen-Nuremberg, Bayreuth, Germany; 5 Center for Medical Image Science and Visualization, Linköping University, Linköping, Sweden

**Keywords:** Gastrointestinal diseases, Computational platforms and environments, Cancer screening, Cancer

## Abstract

In colorectal cancer (CRC), artificial intelligence (AI) can alleviate the laborious task of characterization and reporting on resected biopsies, including polyps, the numbers of which are increasing as a result of CRC population screening programs ongoing in many countries all around the globe. Here, we present an approach to address two major challenges in the automated assessment of CRC histopathology whole-slide images. We present an AI-based method to segment multiple ($n=14$) tissue compartments in the H &E-stained whole-slide image, which provides a different, more perceptible picture of tissue morphology and composition. We test and compare a panel of state-of-the-art loss functions available for segmentation models, and provide indications about their use in histopathology image segmentation, based on the analysis of (a) a multi-centric cohort of CRC cases from five medical centers in the Netherlands and Germany, and (b) two publicly available datasets on segmentation in CRC. We used the best performing AI model as the basis for a computer-aided diagnosis system that classifies colon biopsies into four main categories that are relevant pathologically. We report the performance of this system on an independent cohort of more than 1000 patients. The results show that with a good segmentation network as a base, a tool can be developed which can support pathologists in the risk stratification of colorectal cancer patients, among other possible uses. We have made the segmentation model available for research use on https://grand-challenge.org/algorithms/colon-tissue-segmentation/.

## Introduction

Colorectal cancer (CRC) is the third most commonly occurring cancer in men and the second in women and is expected to affect more than 2.2 million new cases and cause 1.1 million deaths by 2030^1^. The diagnostic pathway of CRC often begins with the detection of cancer in histological samples of polyps or biopsies, identified and acquired during colonoscopy procedure. The detection of polyps is often the result of colorectal cancer *screening*, a program that targets 110 million people a year in Europe, triggering the need for further examination via colonoscopy for about 5% of the participants.

### Histopathology in colorectal cancer diagnosis

**Figure 1:**
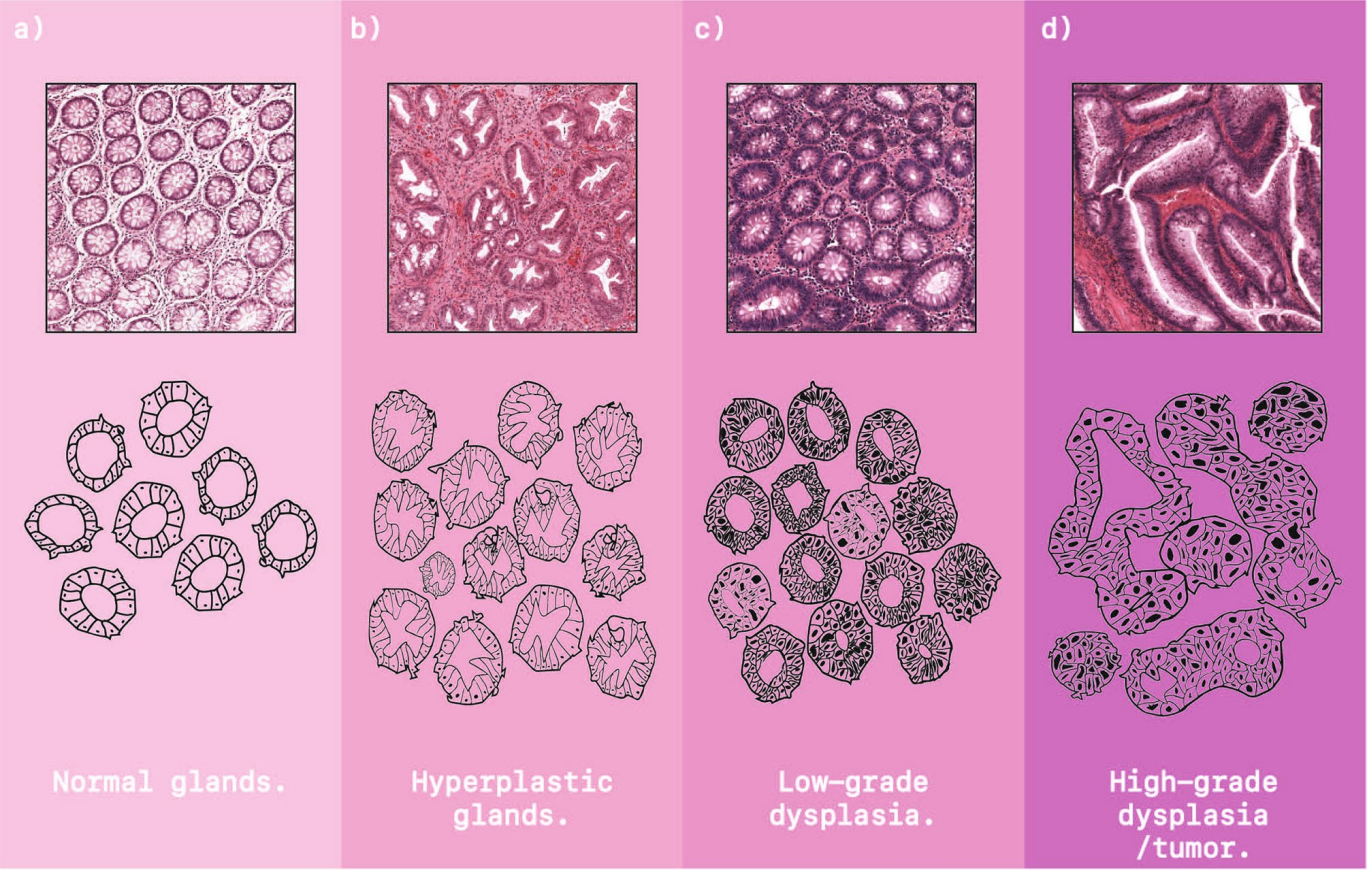
Schematic overview of glandular (de)formation with associated grading class. (**a**) Normal glands; small, organized nuclei and round lumen. (**b**) Hyperplastic gland; small nuclei, saw-tooth like formed lumen. (**c**) Low-grade dysplasia; characterized by unorganized, stacked epithelium cells possibly with enlarged nuclei. (**d**) High-grade dysplasia/tumor; Unorganised fusing glands that oppress the lumen. This figure has been created with Adobe Illustrator 23 https://www.adobe.com/products/illustrator.html and Procreate https://procreate.com/ipad.

In order to diagnose CRC, histopathologists initially *analyze the tissue morphology* of polyps and biopsies to differentiate normal epithelial cells, lying in the usual glandular formation, from epithelial cells in a different configuration that display characteristics associated with the progression towards cancer. This assessment, called *grading*, concerns the degree of glandular arrangement, ranging from normal glands to cancer, with intermediate grades like hyperplasia and dysplasia (see Fig. 1).

When CRC is suspected, an additional tissue characterization step is needed, often performed on surgically resected specimen, to guide the decision on the best therapeutic procedure and patient follow-up. In this context, in recent years several prognostic biomarkers have been introduced and investigated in the field of CRC. Examples are the tumor-stroma ratio^2^, based on the assessment of the tumor region and the tumor-associated stroma; tumor budding^3^, based on the detection of small tumor clusters (up to four tumor cells) at the invasive margin of the tumor; tumor deposits^4^, i.e., a discrete nodules of cancer in pericolic/perirectal fat or adjacent mesentery, based on the assessment of small tumor aggregates located in the adipose tissue.

### On the role of computers in CRC diagnosis

The increasing amount of screening-detected polyps, together with the high percentage of negative (i.e., not containing cancer) samples represents a burden in the diagnostic pipeline, where pathologists have to inspect a large amount of benign tissue. The presence of computer-aided diagnosis systems applied to digital pathology whole-slide images could ease the diagnostic procedure by pre-screening of digitized tissue slides and classifying them based on their clinical outcome, ideally asking pathologists to diagnose only cases containing suspicious lesions. At the same time, the quantification of aforementioned biomarkers often relies on visual estimation of morphological tissue compartments, a task that implicitly carries the subjectivity of pathologists’ opinion. Computers can play a role by assisting pathologists in the detection and recognition of tissue compartments, allowing their objective quantification, towards more reproducible and therefore reliable biomarkers. Two main tasks are common to most of the aforementioned steps in the diagnostic procedure: (1) the detection of tumor cells, to diagnose the presence of the malignancy, and (2) the characterization and detection of multiple tissue compartments, to characterize the morphology of the histology. Examples are regions containing the tumor and its invasive margin, the stroma, as well as regions that might be excluded during biomarker’s assessment, such as the muscle layer, presence of necrotic regions, or healthy glands and related lamina propria.

### Semantic segmentation as a computer aided diagnosis building block

In recent years, Deep Learning methods based on Convolutional Neural Networks (CNN) have been successfully applied to multiple tasks in the medical domain, showing human-level performance in the field of dermatology^5^, radiology^6^, ophthalmology^7^, and pathology^8^. In digital pathology, computer algorithms based on tissue *segmentation* can be used as the main component of approaches to automate the interpretation of tissue morphology. With segmentation, an image is divided into a set of non-overlapping regions, each with its particular shape, border, and semantic meaning. When applied to multiple tissue compartments, i.e., in a multi-class fashion, tissue segmentation can allow to distinguish the tumor from other tissues, to be used for biomarker assessment. Furthermore, the multi-class segmentation output can be used as input to *classification* models that can be trained to make predictions at whole-slide image-level, with possible applications to grading of polyps and biopsies. Kather et al.^9^ was one of the first who used patch classification to segment nine different tissue types. Since they released their dataset publicly, a large corpus of deep learning methods^10^ has been developed to segment CRC using the same nine classes^11–13^. Bustos et al.^14^ also use these 9 classes but use the output of the segmentation model to identify microsatellite instability-status. Others focused on gland segmentation (often including their lumen)^15^, as well as segmenting all their instances, also fostered by the introduction of international challenges such as GLAS^16^ and CRAG^17^. One recent study^18^ used tumor segmentation to predict disease free survival for stage II and III colorectal cancer patients.

### What loss function for semantic segmentation?

Within the medical image analysis as well as the computational pathology community, deep learning models based on an encoder-decoder architecture such as U-Net^19^ are nowadays considered the premier choice for image segmentation^20,21^. During training of deep learning models for segmentation, the parameters of the model are optimized by minimizing the difference, encoded by the *loss function*, between the model’s predictions and the real label from the reference standard. Therefore, the selection of an effective loss function to address the segmentation problem is a relevant yet open question in order to maximize the performance of the trained model. Traditionally, most segmentation models proposed in the computer vision and medical imaging community have used pixel-wise categorical cross-entropy. However, this loss function performs suboptimally in the presence of over- or under-represented classes in the image, causing the over-represented class to dominate the training of the CNN, leading to a biased model. This problem is especially apparent in digital pathology for segmentation tasks where small tissue compartments (e.g., erythrocytes) need to be segmented correctly next to larger components (e.g., muscle). To address this, some authors have proposed the use of weights or penalty terms to the categorical cross-entropy loss function^22^. In^12^, the authors proposed the use of a *focal loss*, which adds a term to reduce the contribution of easy examples to improve CNN focus on the difficult examples while others^23^ have made modifications to counteract undesirable effects of noise in the reference standard. In conjunction with an increasing use of the *Dice score* and the *Jaccard index* as a metric to assess model performance, differentiable approximations of these two metrics have recently been formulated, known as surrogates, such as *soft-Dice*^24^, *soft-Jaccard*^25^ and *Lovasz-softmax*^26^; their differential property makes them usable as a loss function for model training.

### Our contribution

In this paper, we introduce several contributions within the context of computed aided diagnosis of colorectal cancer. First, we introduce a deep learning based algorithm for multi-class semantic segmentation addressing fourteen different tissue types in whole-slide images of colorectal cancer, including not only the primary cancer-associated epithelial and stroma classes but also some other more peripheral tissue types. The selection of these fourteen classes allows us to provide a detail characterization of the colorectal tissue at hand, also beyond the detection of tumor regions. Although not shown in this paper, the same segmentation model could be used as a building block for a broad range of applications such as identifying peri-neural invasion^27^, quantification of the tumor-stroma ratio, as we did in previous research^28^, or move into quantification of multiple types of cells such as immune cells in different tissue compartments, which is the base for ongoing research on computational biomarkers based on spatial biology. We demonstrate the robustness of this segmentation algorithm when applied to multi-centric data, acquired from different pathology laboratories and digitized with different scanners.

We also show the effectiveness of the segmentation algorithm on publicly available datasets such as GLAS^16^ and CRAG^17^. Second, we dissected the design of convolutional neural networks for segmentation and investigated the particular design choice of the loss function. For this purpose, we considered a pool of loss functions proposed and used in the computer vision community to address limitations of cross-entropy loss and improve performance in semantic segmentation problems. We selected four representative loss functions: (1) Categorical Cross-entropy loss, (2) Focal loss^29^, (3) Bi-tempered loss^23^ and (4) Lovasz-softmax loss^26^. We report an experimental comparison using a multi-centric dataset of CRC cases from five different centers, as well as two publicly available datasets. To the best of our knowledge, this is the first time that such a research question is addressed within the context of computational pathology and that such a comparison is performed. Third, we took the best performing segmentation model and use it to develop a computer aided diagnosis system to automate risk classification of CRC polyps and biopsies from population screening addressing four of the main diagnostically relevant categories, namely (1) high-risk (tumor and high-grade dysplasia), (2) low-grade dysplasia, (3) hyperplasia and (4) benign conditions. We validated this approach on an external dataset of polyps and biopsies from > 1000 patients. Korbar et al.^30^ directly addressed the problem of automatic classification of colon biopsies in whole-slide images, but solely focusing on five non-neoplastic conditions, namely hyperplastic, sessile serrated, traditional serrated, tubular, and tubulovillous/villous. Other authors^10^ have focused on the classification of regions of interest (i.e., patches) of WSI as containing tumor or non-tumor tissue without attempting to assess entire colon resections or biopsies.

With the presented work we cannot only automate risk classification but the proposed method could also serve as the foundation for a wide range of applications, including those for diagnosis (biopsy classification, tumor size identification), research on the tumor micro-environment (tumor-stroma ratio, peri-neural invasion), and histological features for prognosis (tumor shrinkage^31^). The presence of computer-aided diagnosis systems applied to digital pathology whole-slide images could support pathologists in their diagnostic procedure. Depending on the characteristics, performance, robustness, and generalizability of such a system, several scenarios of integration of AI in diagnostics could be envisioned. One scenario could leverage AI to pre-read cases, extracting relevant information to pre-fill the report, and show the results to the pathologist, who just needs to check the case, the results and sign-off. Another scenario could leverage AI to pre-score cases based on their risk and use this to present cases to pathologists in some order of importance or diagnostic urgency. In all cases, these systems have the potential and the aim to support pathologists in spending less time on diagnostics while maintaining its quality, and therefore also reducing healthcare costs.

**Figure 2:**
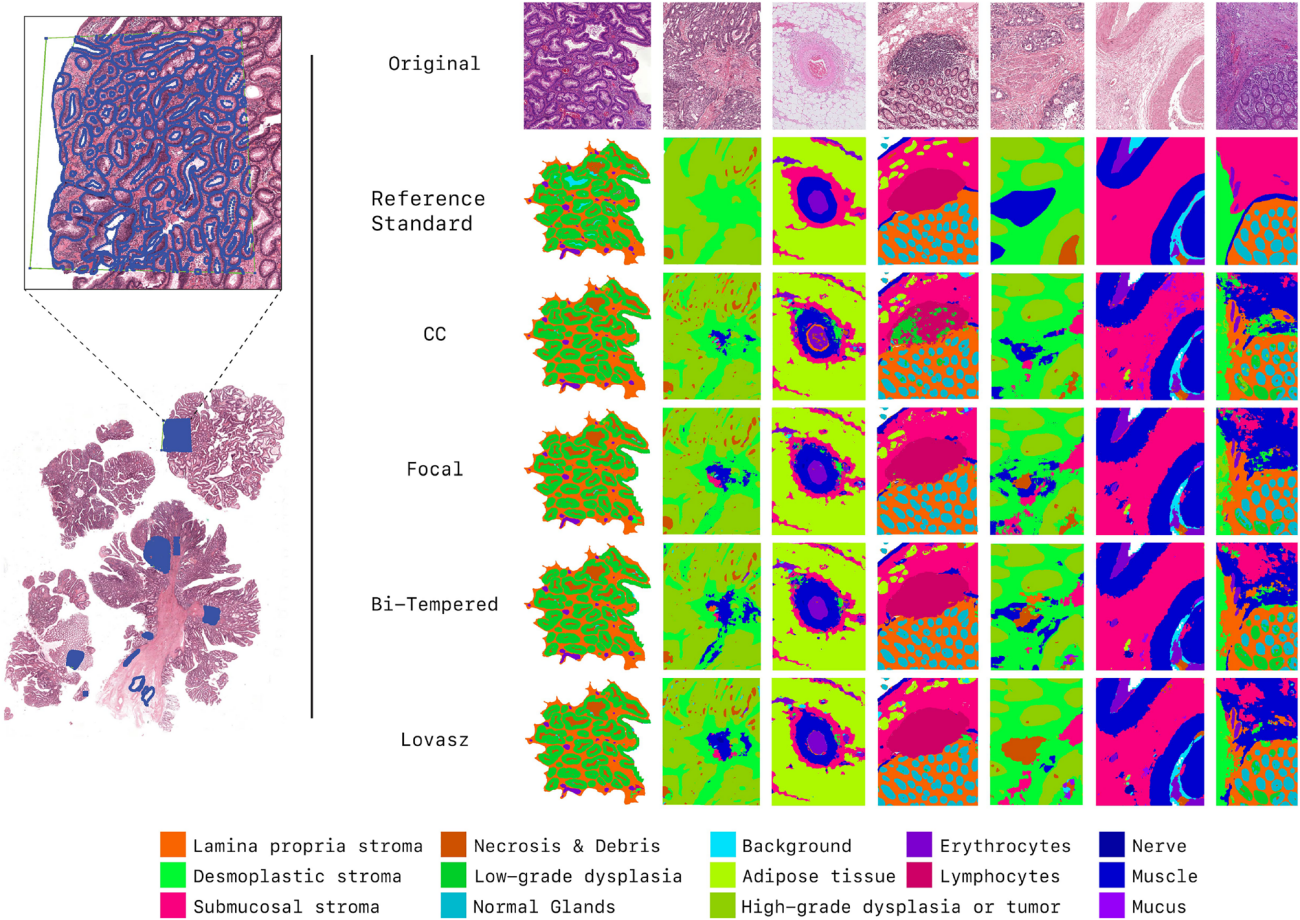
Left; example of manual annotations. Right; segmentation output on the private test-set. Every image shown is from another institute from the test-set. This figure has been created with Adobe Illustrator 23 (https://www.adobe.com/products/illustrator.html).

This paper goes as follows. The next chapter describes, by nature and origin, the data used for the development of the multi-class segmentation model and of the independent data set that was used for the validation/evaluation of the 4-class classification model. In section “Multi-class segmentation and loss functions” special attention is paid to the network architecture and the four loss functions mentioned above are described in more detail on their intended effects and limitations. Finally, section “Experimental results” discusses how the computer aided diagnosis system is constructed for the performance of the -here secondary or derived- risk classification task. We present the findings of this study with considerations and recommendations in Sections “Discussion” and “Conclusion”.

## Materials

In this study, we used multiple datasets of digital pathology images from polyps, biopsies and surgical resections, which were used for training, validation and testing of the segmentation model, as well as for training, validation and testing of the classifier for automated risk assessment of polyps and biopsies. All datasets are described in the next sections. An overview of all datasets used can be found in Table 3.

**Figure 3:**
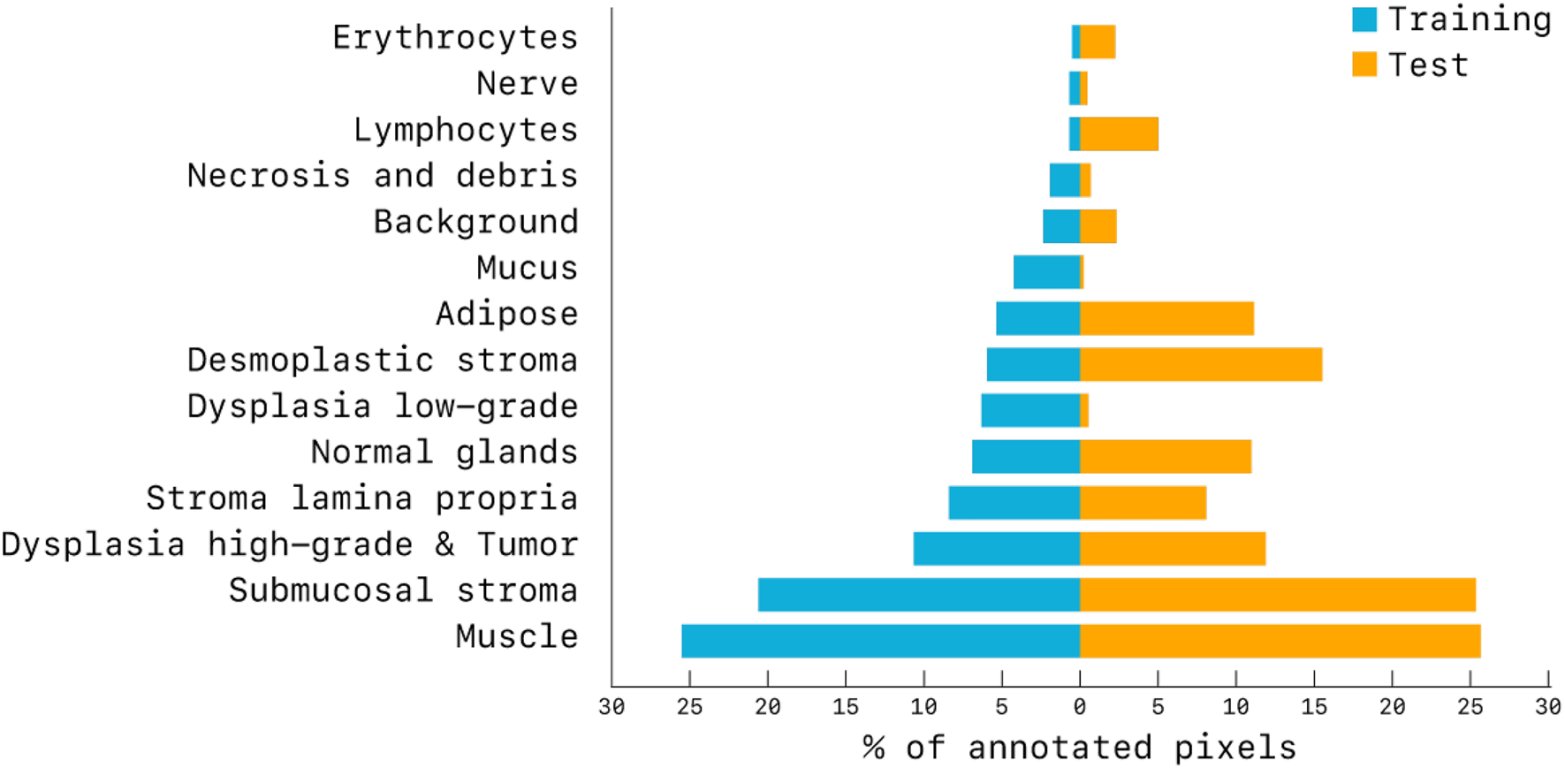
Distribution of the annotated pixels of the training set ($D^{train}_{seg}$) and test set ($D^{test}_{seg}$). This figure has been created with Microsoft Excel (https://www.microsoft.com/en-us/microsoft-365/excel) and Adobe Illustrator 23 (https://www.adobe.com/products/illustrator.html).

### Tissue segmentation dataset

The first dataset ($D_{seg}$) was used for development and validation of the segmentation algorithm. A total of *n* = 79 formalin-fixed paraffin-embedded tissue samples, including surgical resections and biopsy specimen of colorectal cancer patients were collected in a multi-centric fashion from four different medical centers in the Netherlands and one medical center in Germany. All slides were stained with H&E in the pathology laboratory of each medical center, resulting in a large variety of staining. Furthermore, all slides were digitized using three different types of digital pathology scanners, resulting in a substantial tissue appearance variation in whole-slide images. The Pannoramic P250 Flash II scanner (3D-Histech, Hungary) scanner was used to digitize *n* = 62 slides provided by Radboud University Medical Center (Nijmegen, Netherlands), *n* = 4 slides provided by Eindhoven Medical center (Eindhoven, Netherland), and *n* = 5 slides provided by Utrecht University Medical Center (Utrecht, Netherland). The IntelliSite Digital pathology slide scanner (Philips, the Netherlands) was used to digitize *n* = 5 slides provided by the Leiden University Medical Center (Leiden, Netherland). The NanoZoomer 2.0 HT scanner (Hamamatsu, Japan) was used for digitizing *n* = 3 slides provided by the University of Bayreuth (Germany). All slides were scanned at a spatial resolution of 0.24 μm/px.

In each WSI, regions of interest covering a broad range of different tissue morphology and tissue components were manually selected. In each region of interest, one pathologist and two trained medical/technical analysts manually annotated and checked pixels as belonging to one of the 14 following categories: (1) normal glands, (2) low-grade dysplasia, (3) high-grade dysplasia/tumor, (4) submucosal stroma, (5) desmoplastic stroma, (6) stroma lamina propria, (7) mucus, (8) necrosis and debris, (9) lymphocytes, (10) erythrocytes, (11) adipose tissue, (12) muscle, (13) nerve, (14) background. Each region was exhaustively annotated, meaning that all pixels within the region of interest were labeled, accurately delineating interfaces of different tissue compartments. Annotations were made using the in-house developed open-source software ASAP https://github.com/computationalpathologygroup/ASAP. Visual examples of manually annotated regions are depicted in Fig. 2. An overview of the proportion of annotated classes compared to the total amount of annotations can be found in Fig. 3.

From the $D_{seg}$ dataset, a set of *n* = 52 WSIs from a single center (Radboud University Medical Center) and their annotations were randomly selected and used to define a single-center training set ($D^{train}_{seg}$, *n* = 40) to develop segmentation models and a validation set ($D^{val}_{seg}$, *n* = 12) used to optimize hyperparameters during training. The rest of WSIs ($D^{test}_{seg}$, *n* = 27) and their manual annotations was used as a multi-centric test-set. Note that among the *n* = 27 test slides, *n* = 10 were originated in the same medical center as the training set, and the rest (*n* = 17) were originated in different medical centers, and partly scanned using different scanners.

### Biopsy classification dataset

A dataset $D_{cls}$ of colon biopsies resected from *n* = 1054 patients was collected from the digital archives of the Cannizzaro hospital (Catania, Italy). For each case, digital pathology whole-slide images of H&E stained tissue section as well as the related pathology report was collected. Based on the conclusion of the report, each tissue sample was assigned by an export to a single label, corresponding to the most clinically relevant finding diagnosed by the pathologist. For this purpose, ”high-risk” (including tumor and high-grade dysplasia) was considered as the category with the highest relevance (n = 292 cases), followed by low-grade dysplasia (n = 693), followed by hyperplasia (n = 36) and finally by all other (non informative, n = 33) cases, such as but not limited to normal cases. When multiple findings were present in a single slide, e.g., both hyperplasia and high-grade dysplasia, the label with the highest associated risk was appointed to the case. This set of cases was used to develop and validate a prediction model that processes features derived from the segmentation map of each biopsy, and to automatically classify each biopsy into one of the aforementioned categories. All slides were scanned with a Aperio AT2 (Leica Biosystems) at a spatial resolution of 0.24 μm/px.

**Figure 4:**
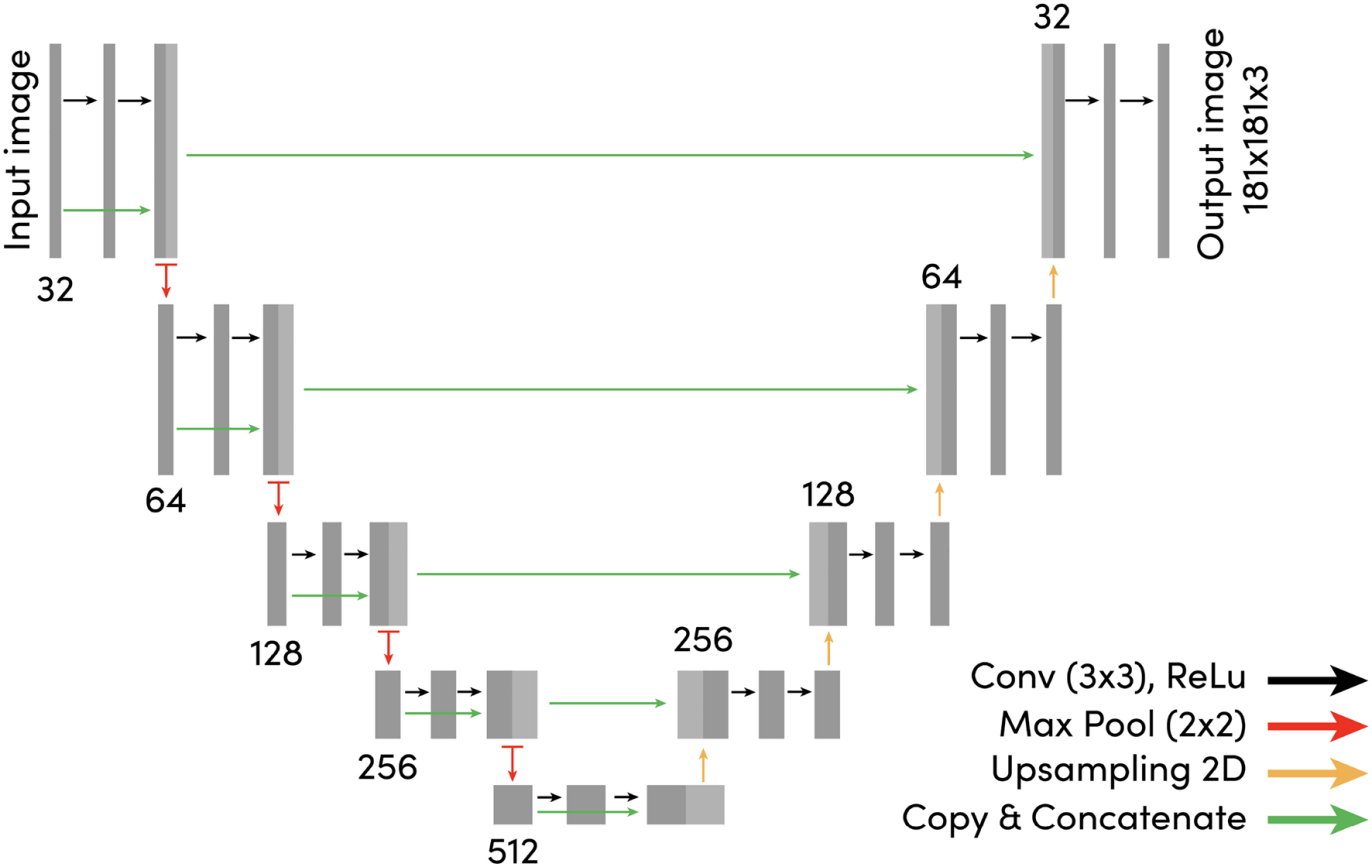
Overview figure of the used U-Net architecture. The light-gray boxes represent copies of the feature maps. Each dark-gray box represents a multi-channel feature map. The input size is shown on the left of the box, and the number of channels on top.The arrows denote the different operations. This figure has been created with Adobe Illustrator 23 (https://www.adobe.com/products/illustrator.html).

### Ethical approval

The use of the fully anonymized data for training and validating the segmentation network has been approved by the Ethical Committee of the Radboud University Medical Center (2015-1637). During their cancer treatment, patients were informed that left-over tissue material could be used for research, and at that time, they had no objections to such use and provided their informed consent. The Ethical Committee approved the usage for training and validation of the biopsy classifier of the Cannizzaro Hospital (approval number 4428, 12/12/2018). This research was performed following the Declaration of Helsinki.

## Multi-class segmentation and loss functions

In this section, we detail the CNN model used for automated multi-class tissue segmentation based on the U-Net architecture, followed by the training procedure. Successively, we detail the different loss functions considered and compared in this work, and we provide a short description of each loss function’s main characteristics. Finally, we describe the prediction model developed for the automated classification of biopsies.

### Segmentation model architecture and training

We designed our segmentation model based on the original formulation of the U-Net architecture; an overview of the used U-Net model is depicted in Fig. 4. As done in the original U-Net model, we have doubled the number of filters after every max-pooling layer, but we started with a lower number of filters in the first layer (n = 32). Additionally, inspired by the ResNet^32^ approach, we introduced additional skip connections within every set of convolutional layers (which we call a U-Net block) before pooling, where the input of the U-Net block is concatenated with the last feature map produced by the block itself. This approaches was already used by others^20^, and in this work, we observed experimentally that adding these skip connections allowed better flow of the gradients. Finally, we replaced transposed convolutions with nearest neighbor up-sampling operations followed by a 2 x 2 convolution layer in the expansion path.

Besides the loss function, the training settings and procedure were kept identical between the different experiments. The input was a RGB patch of 512 × 512 px sampled from whole-slide images in $D_{seg}$ at a pixel size of 1 μm (10 × magnification). The training procedure involved optimizing the multinomial logistic regression objective (softmax), using Adam^33^ optimizer with momentum. Momentum values were identical to the original U-Net paper. During training data augmentation was applied to input patches by random flipping, rotation, elastic deformation, blurring, brightness (random gamma), stain, color, and contrast changes. An adaptive learning rate scheme was used, where the learning rate was initially set to 1e−4 and then multiplied by a factor of 0.5 after every 20 epoch if no increase in performance was observed on the validation set. The weights of the network were initialized as proposed in^34^. The mini-batch size was set to 5 instances per batch, the networks were trained for a maximum of 500 epochs, with 300 iterations per epoch. Training of the networks was stopped when no improvement of the validation loss was found for 50 epochs. The output of all networks is in the form of *C* likelihood maps. The pixel-wise arg-max was taken to obtain a final segmentation output.

**Figure 5:**
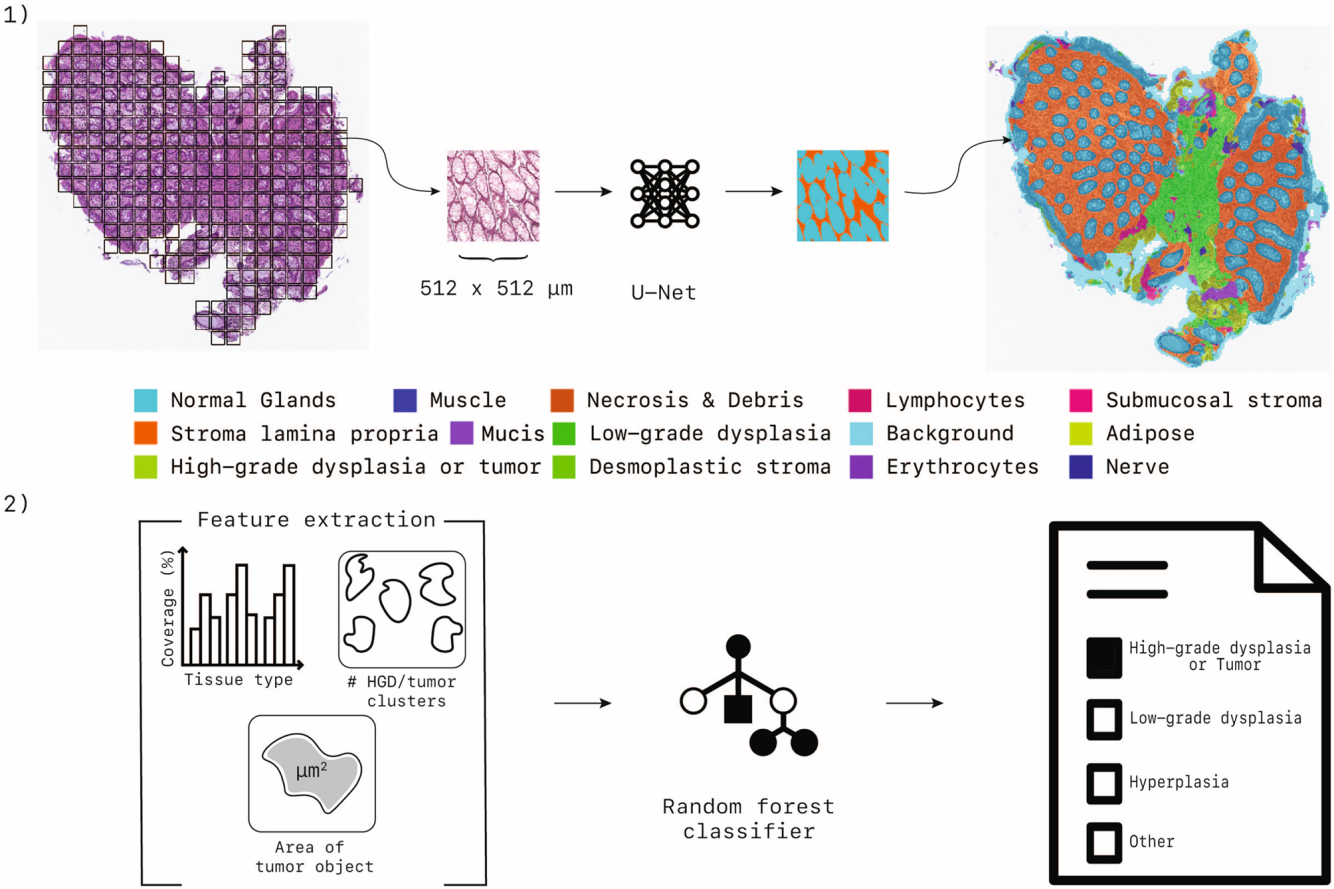
(**1**) Segmentation process; An encoder-decoder segmentation model based on convolutional neural networks processes colorectal tissue tiles with a size of 512 × 512 μm and segments up to 14 different tissue types. (**2**) From the segmentation map at slide level, we extract a set of features: (a) the normalized histogram of all tissue types, (b) the number of high-grade dysplasia/tumor clusters, (c) the average, minimum, and maximum size of these clusters. These features are processed by a random forest classifier that gives the final classification. This figure has been created with Adobe Illustrator 23 (https://www.adobe.com/products/illustrator.html).

### Analysis of loss functions for multi-class tissue segmentation

#### Categorical cross-entropy

In the context of image segmentation, the cross-entropy loss, also known as softmax loss, has been largely used both in binary and multi-class problems, referred to as categorical cross-entropy (CC) loss in the presence of a multi-class problem. This loss compares how well the probability distribution output $\hat{y}$ of the final softmax layer of a deep neural network matches the value of the ground-truth data *y*. In segmentation tasks, this is done pixel-wise for every pixel in the image. Typically, the sum or mean of all values together gives the final single value for the entire input image. The categorical cross-entropy loss is computed as

1 $$\begin{aligned} loss = - \sum _{i}^{C} y_i \log \hat{y}_i, \end{aligned}$$

where $y_i$ and $\hat{y}_i$ are the ground truth and the softmax output of the model for each class *i* in *C*. Although being one of the most common loss functions, it is known to perform poorly under certain conditions, such as class imbalance or noise in the labels of the dataset.

Many authors have been concerned with the problem of imbalanced classes: negative examples significantly outnumber positive examples, and the huge number of background examples (or easy-negative examples) overwhelms the training. Training a network with the categorical cross-entropy loss on an imbalanced dataset can lead to a network that is biased towards the data-dominated classes. This becomes especially apparent in segmentation tasks when small objects need to be segmented. Because of the low pixel count, these small objects don’t have a large contribution to the loss function.

#### Focal loss

To overcome this issue, Lin et al.^29^ proposed the Focal loss:

2 $$\begin{aligned} loss = - \sum _{i}^{C} \alpha (1 - \hat{y}_i)^\gamma y_i \log \hat{y}_i. \end{aligned}$$

Presented initially to improve single-shot detection networks (such as YOLO^35^, Single Shot Detector^36^, and Retinanet^29^), it has shown to work for segmentation problems as well^37,38^. This loss modifies the cross-entropy loss, so objects/pixels that are ’easy’ to classify or are present abundantly are weighted lower, resulting in a smaller loss value. Objects/pixels that are more difficult to classify are weighted heavier, resulting in a higher loss value. How much up- or down-weighted the values are, is determined with two additional hyperparameters (α and γ). The authors have tested different values of the two hyperparameters in the original paper and suggested two optimal values. Given the scope of this paper, we have simply adopted both proposed values here.

#### Bi-tempered loss

The presence of small errors, flaws or defects (noise) in the dataset, for example as a result of small inaccuracies in the annotation can hamper the quality of a segmentation output substantially. In such scenarios, the categorical cross-entropy loss value can grow without boundary as these outliers can be far away from any decision boundary: the model is penalized by noise in the dataset. To compensate for this, the network stretches its decision boundary, resulting in a less robust model. To counteract this effect, Amid et al.^23^ introduced the Bi-tempered loss function. They propose two changes to the default softmax categorical cross-entropy. First they replace the softmax output with a heavy tailed softmax, that acts as a form of ’label smoothing’. The replacement softmax function is given by:

3 $$\begin{aligned} \begin{aligned} \hat{y}_i = \exp _{t_2} (\hat{a}_i - \lambda _{t_2}(\hat{a})), with \ \lambda _{t_2}(\hat{a}) \in {\mathbb {R}} \end{aligned} \end{aligned}$$

such that $\sum _{j}^{C} \exp _{t_2} \left( \hat{a}_j - \lambda _{t_2}(\hat{a}) \right) = 1$, where *a* are the activations from the final layer of the network, and $a_i$ is the output for class *i*, $t_2$ is a hyperparameter. The second change they make is by replacing the entropy function with a tempered version, given by:

4 $$\begin{aligned} loss = \sum _{i}^{C} \left( y_i(\log _{t_1} y_i - \log _{t_1} \hat{y}_i) - \dfrac{1}{2-{t_1}}(y_i^{2-{t_1}} - \hat{y}_i^{2-t_1}) \right) . \end{aligned}$$

The two hyperparameters determine how heavy tailed the functions become. When both hyperparameters ${t_1}$ and ${t_2}$ are set to 1, the standard logistic loss is recovered. We have not tested this option either, but have kept the default values proposed by the authors.

#### Lovasz loss

Optimization for cross-entropy entails a problematic relationship between the learning optimization objective (the loss) and the end target metric. Therefore, recent works in computer vision have proposed soft surrogates to alleviate this discrepancy and directly optimize the desired metric, either through relaxations (soft-Dice, soft-Jaccard/Intersection over Union) or submodular optimization (Lovasz-softmax). Dice loss is based on the Sorensen-Dice coefficient, which attaches similar importance to false positives and false negatives, and is less sensitive to the data-imbalance problem. Instead of a pixel-wise approach, the Dice or Intersection over Union (IoU) calculates the similarity between two samples of the same class resulting in a value between 0 and 1. The scores are often averaged over all classes for multi-class problems, resulting in the mean-Dice or mean-IoU. Advantages of this index compared to per-pixel losses are scale invariance and appropriate counting of false negatives, although it tends to favor large objects over small objects. To deal with this issue^26^ combined the Lovasz hinge with the IoU resulting in a loss function that seems to focus both on small and large objects:

5 $$\begin{aligned} loss = \dfrac{1}{\mid C \mid } \sum _{i}^{C} \overline{\Delta _{J_c}} \left( {{\textbf {m}}}(i) \right) , \end{aligned}$$

where $\overline{\Delta _{J_c}}$ is the Lovasz hinge of the IoU and ${{\textbf {m}}}(c)$ the class probabilities for class *c*.

#### Dice loss

In a previous study^39^, we have studied and tested a number of different class balancing methods. The Dice loss proved difficult to implement in combination with our preferred class balancing method. We therefore chose to maintain/re-apply the relevant class balance method in this study and not to include the Dice loss in the comparative study. We will use the Dice metric however, to evaluate the models performance.

## Biopsy classification

An additional classification step is needed to obtain a single label per slide from a segmentation output. To stay as close as possible to the segmentation output, without introducing complex models, we have opted for a random forest classifier, combined with five easy interpretable features. We extracted the number of pixels of every class in the segmentation output (histogram) and normalized it by dividing the histogram by the total number of segmented pixels as the first feature. The normalized histogram does not tell us anything about the spatial distribution of, for example, the tumor epithelium, although that distribution may matter. Since tumor mostly comes in clusters we, in addition to the normalized histograms (1), used connected components to obtain the number of tumor clusters per case (2), along with the average (3), min (4), and max (5) size of all clusters. We noted that the segmentation model sometimes falsely segments small clusters as tumor. Therefore, we excluded all clusters smaller than 30 μm^2^ from the segmentation output to correct these mistakes. The exclusion cut-off value of 30 μm^2^ was found empirically. Using the aforementioned features we trained a random forest with 1000 decision trees to classify a biopsy as either (1) high-risk (tumor and high-grade dysplasia), (2) low-grade dysplasia, (3) hyperplasia and (4) benign. A schematic overview of the approach is depicted in Fig. 5, a block diagram of the entire approach can be found in Fig. 8.

The random forest was trained in a five-fold cross-validation setup on dataset $D_{cls}$. Before training, all feature values were normalized to have zero mean and scaling to unit variance. The same normalization parameters were applied to the test-set before running the classifier. Per fold, a random forest was trained using four of the five folds as training data. The performance of the model is validated on the remaining part that was left out during training. We evaluated the performance of the trained model with a 1-vs-all Receiver Operating Characteristic (ROC) analysis.

## Experimental results

### Segmentation performance on a multi-centric dataset

We evaluated the different loss functions’ effect on 999 non-overlapping tiles of 512 × 512 μm, extracted from a total of 27 WSI from 5 different centers, hereafter referred to as $D^{test}_{seg}$. The non-overlapping tiles were extracted from 165 manually annotated regions with an area between 0.05 mm^2^ and 4.08 mm^2^. The Dice-score was selected to evaluate the performance of the different models. As the Dice-score is for binary problems, we calculate an individual, (class) Dice-score as well as a mean overall Dice-score per model (see Table 1).

The model trained with the Lovasz-softmax loss achieves the overall best performance with a Dice-score of 0.72, although this score is well matched by the Bi-tempered loss model (Dice-score = 0.71). Models trained with the categorical cross-entropy and Focal loss score slightly lower (Dice-score = 0.69). The Wilcoxon signed-rank test shows there is no significant difference between the overall scores of the loss functions.

Although the differences between the overall scores of models trained with different loss functions are marginal, there are sometimes notable differences for the results per class. For example, the Bi-tempered loss outperforms the other loss functions on segmenting low-grade dysplasia. Results are depicted in Fig. 2.

The overall Dice-scores per test-center for the centers in dataset $D^{test}_{seg}$ are in line with the overall average Dice-scores. However, in one of the external test-centers, all trained models show suboptimal performance in the presence of submucosal stroma, presenting a drop from an averaged Dice-score of 0.54 overall to 0.28. Further investigation of this test-center shows that these slides have a very dark H &E staining that could harm the performance of the networks. An overview of all Dice-scores per center can be found in Fig. 9.

**Figure 6:**
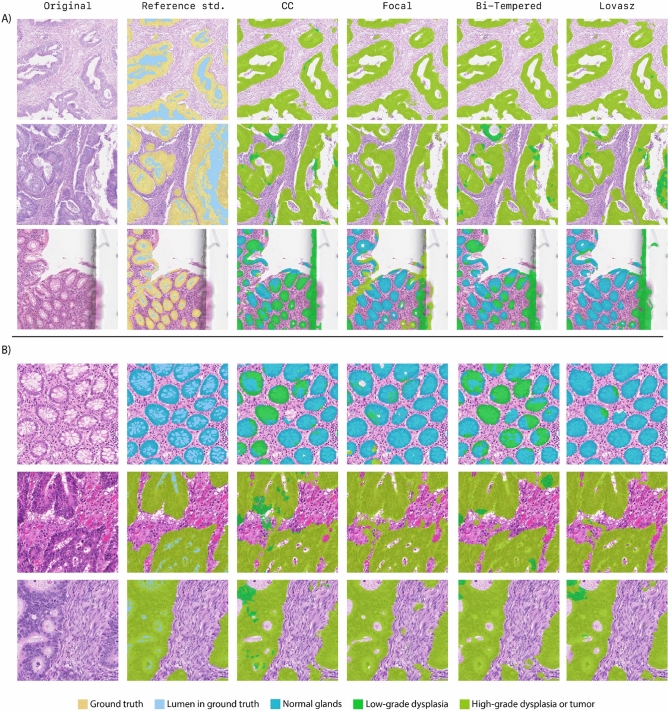
Segmentation output on the (**A**) CRAG and (**B**) GLAS challenge. The F1-scores are calculated on the reference standard images where the background (light-blue) has been removed. Note, because the CRAG challenge is a binary segmentation task, we marked the epithelium in the reference standard with a single color. This figure has been created with Adobe Illustrator 23 (https://www.adobe.com/products/illustrator.html).

**Figure 7:**
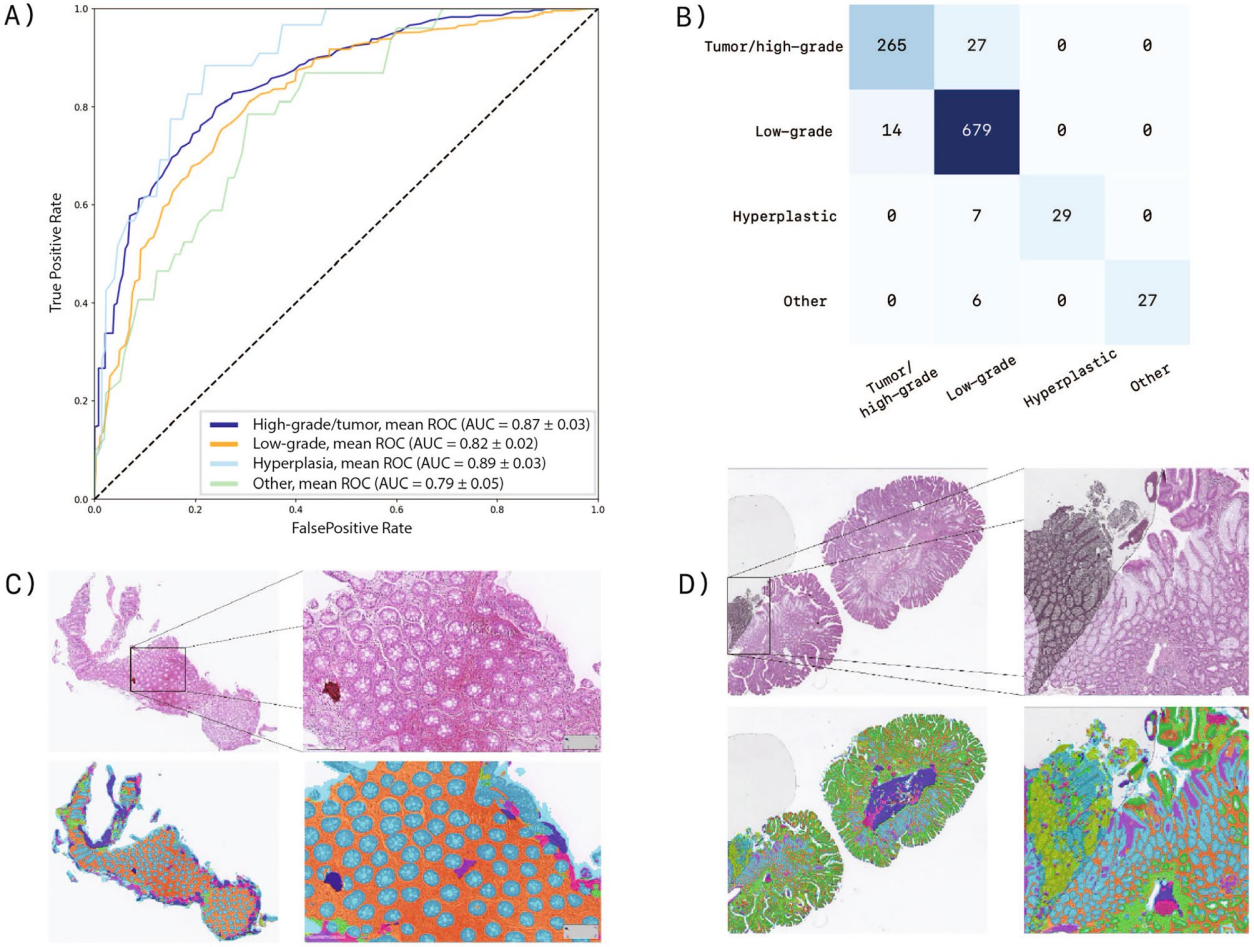
(**A**) ROC-curves of the random forest classifier. (**B**) Confusion matrix of the biopsy classifier. (**C**) Segmentation output of a healthy fragment. (**D**) Segmentation result that fails due to air bubble on the glass slide. This figure has been created with Adobe Illustrator 23 (https://www.adobe.com/products/illustrator.html).

**Table 1:** Dice-scores of different loss functions on the entire private test-set.

	Categorical cross-entropy	Focal	Bi-tempered	Lovasz
Tumor	**0.89**	0.87	0.87	0.83
Desmoplastic stroma	**0.69**	0.54	0.64	0.58
Necrosis and debris	0.46	**0.49**	0.47	0.45
Lymphocytes	0.61	0.82	0.82	**0.84**
Erythrocytes	0.62	0.66	0.65	**0.68**
Muscle	0.81	0.84	**0.85**	0.82
Submucosal stroma	0.50	**0.58**	0.55	0.54
Adipose tissue	0.85	0.85	**0.89**	0.86
Mucus	**0.64**	0.46	0.60	0.62
Nerve	0.63	0.54	0.69	**0.83**
Normal glands	0.85	0.87	0.82	**0.88**
Lamina propria	**0.88**	0.86	0.87	0.82
Background	0.46	0.45	0.36	**0.51**
Dysplasia low grade	0.80	0.78	**0.88**	0.77
Average	0.69	0.69	0.71	**0.72**

Significant values are in bold.

**Table 2:** F1-scores of different loss functions on the public CRAG and GLAS datasets.

	CRAG		GLAS	
	w lumen	w/o lumen	w lumen	w/o lumen
Categorical cross-entropy	**0.68**	**0.77**	**0.79**	**0.80**
Focal	0.66	**0.77**	0.76	0.78
Bi-tempered	0.54	0.69	0.71	0.75
Lovasz	**0.68**	0.76	**0.79**	**0.80**

Significant values are in bold.

Results are computed both when the luminal area (w lumen) is included as well as removed (w/o lumen).

### Segmentation performance on the CRAG and GLAS datasets

We applied our segmentation models to the public datasets of the CRAG and GLAS challenge. The CRAG challenge’s goal is to segment all glands (epithelium tissue, lumen included), either with or without pathological change. As our network also differentiates between different epithelial conditions, we combined our classes ’normal epithelium’, ’low-grade dysplasia’, and ’high-grade dysplasia/tumor’, for the purpose of comparison with this dataset. Because the GLAS challenge distinguishes between benign and malignant epithelium, we combined our classes ’normal epithelium’ and ’low-grade dysplasia’ for comparison in that context. For these comparisons, we further relabelled the lumen (pixels with a mean RGB value higher than 240) as background in both public datasets since our networks are designed to segment such regions separately. In Table 2, we report both results with and without lumen, to show the impact of this on our models’ performance.

The results on the GLAS and CRAG datasets deviate from the results on the multi-centric test-set. The categorical cross-entropy and Focal losses show increased performance on the CRAG dataset (without lumen) with a Dice-score of 0.77, and Lovasz-softmax loss performs slightly worse here (Dice-score = 0.76), but the aforementioned three loss functions perform almost equally well on the GLAS set (without lumen). Remarkably, the Bi-tempered model achieves the worst results on the two public data sets with Dice-scores of 0.69 and 0.75 on CRAG and GLAS, respectively.

Without the removal of the lumen, all networks obtained lower Dice-scores. On the CRAG dataset, all networks show Dice-scores that are on average 0.1 lower. The categorical cross-entropy and Lovasz-softmax loss perform best with a Dice-score of 0.68, followed closely by the Focal loss with a Dice-score of 0.66, the Bi-tempered loss obtained a Dice-score of 0.54. On the GLAS dataset, the differences with and without lumen are less evident. The categorical cross-entropy, Focal, and Lovasz-softmax loss obtain almost equal Dice-scores of 0.79, 0.76, 0.79, respectively. The Bi-tempered loss achieves a Dice-score of 0.71. The Dice-coefficients are shown in Table 2. Visual results on Glas and CRAG data can be found in Fig. 6.

### Evaluation of the colon biopsy classifier

Since the network trained with the Lovasz-softmax loss obtained the overall best Dice-scores and seemed to be the most stable across all different classes, this model was selected for the biopsy classification. One thousand fifty-four biopsies, including polyps, were collected from the Cannizzaro hospital’s pathology department in Catania (Italy) and were processed by the model trained with the Lovasz-softmax loss. We collected the normalized histogram, the total number of tumor clusters, average/min/max size of all tumor clusters, and every tissue-fragment within the WSI from the segmentation output. Five-fold cross-validation was used to train and validate the random forest classifier. The dataset was randomly split into five class-balanced folds. We report the aggregated results on the left-out cases. Before training, all features were normalized to have zero mean and scaled to unit variance. The same statistics learned from the training set were applied to the test-set before using the classifier. A WSI can contain multiple sections of a biopsy. Consequently, it is not guaranteed that all tissue fragments contain tissue that have the same risk class, for example, one fragment can contain cancer while the rest only contains normal tissue. Therefore, all WSI tissue fragments are processed; the worst grade, i.e. the fragment with the worst classification, is then adopted as the final slide label. As shown in Fig. 7 we found an one-vs-all AUC of 0.87 ($\pm 0.03$), 0.82 ($\pm 0.02)$, 0.89 ($\pm 0.03)$, and 0.79 ($\pm 0.05)$ for the classification of high-grade dysplasia/tumor, low-grade dysplasia, hyperplasia, and other respectively. Misclassification of healthy tissue by labeling it as tumor tissue is worse than mislabeling healthy tissue as hyperplastic tissue. Therefore, we used the quadratic weighted kappa to evaluate the classifier’s results. An overall kappa score of 0.91 was reached. The confusion matrix for all cases and some examples of the segmentation output of the biopsies can be found in Fig. 7.

## Discussion

When developing a deep learning model, several components determine the quality of the final result. This paper has set out to determine the effects of different loss functions in a 14-class semantic segmentation task. We selected four different state-of-the-art loss functions, each with its own characteristics, and trained them on WSI’s of a single centre. The four models’ performance on all fourteen classes can best be judged on the private test-set. Although the differences are not significant, the Lovasz-softmax performs best overall on the segmentation task at hand. The score of the Bi-tempered loss is slightly lower and the same applies to the categorical cross-entropy and focal loss. Compared to the categorical cross-entropy, the focal loss shows added value neither on the private set, nor on the public datasets. It is conceivable that the potentially positive effect of the focal loss is lost with a large/larger number of classes. When the number of classes is small a higher and -with two classes- even highest degree of class imbalance can occur, what is the intended type of scenario for this function.

The Bi-tempered loss almost equals the performance of the Lovasz-softmax loss on the private test-set and distinguishes itself positively from both the categorical cross-entropy and the focal loss on this set. We note that this model mainly underperforms on the background class, although all networks seem to perform less well with this class, in addition to the necrosis class. It achieves relatively good dice scores on the low grade and high-grade dysplasia (epithelium) classes, but falls back on the healthy epithelium class. We see this effect in particular in the segmentation output on the images of the other centers (see Fig. 9, Test-Center 2/3/4/5) and therefore were inclined to attribute it to stain variations in certain classes here. The Bi-tempered loss is performing worse on the public test-set however. Images from the Bi-tempered results on the public sets show over-segmentation of the high-grade dysplasia/tumor classes, which can explain these results. Both the Focal and the Bi-tempered loss have two hyperparameters that can influence the overall quality of the networks. For this study, we used the values that the authors of the original papers recommended. Further improvements can be made by searching for the optimal parameters but this falls out of this paper’s scope. The Lovasz-softmax loss also delivers a good performance on the public test-sets and seems to benefit from its relative scale insensitivity in this segmentation task. This also results in an overall smoother representation of the output compared to the other loss functions whose images look a bit grainier. To the eye, border definitions look clearer in the WSI’s processed with the Lovasz loss compared to the other networks. Although there is no significant difference between the metric sensitive Lovasz-softmax loss and the other losses on the various datasets, the Lovasz shows a good and stable prediction quality on the private and public datasets as well, with the convenience that no additional hyperparameter tuning is required. This result is in line with^40^, who not only demonstrate the mathematical superiority of multiple metric-sensitive solutions compared to the categorical cross-entropy (inspired) loss functions, but also substantiate this with empirical research.

In the design of the classification model, we have chosen to stay as close as possible to the result of the segmentation network, assuming that the output of an accurate segmentation model, next to being fully interpretable by pathologists, needs little engineering to generate discriminative features to stratify the risk of colon biopsies. For this reason, the relative amount of tissue per class (class histogram) has been introduced as a first feature. Because this factor lacks spatial distribution information, we decided to include such information for the epithelium class ’tumor’ in the model, in order to be able to more accurately separate this class from related (epithelial-) classes. To this end, we selected the number of tumor clusters, the minimum, maximum and average cluster size as an additional feature. We have applied our segmentation & classification model to 1054 biopsies. We compared the class attributions obtained per network system and per pathologists on this series of biopsies. We performed an extensive error analysis on the mismatch cases with the involvement of pathologists. This analysis shows that roughly forty percent of the classification errors can be traced back to a faulty output of the segmentation model. In overstained specimen, the dark regions are sometimes incorrectly identified as tumor, while that tissue should have been given the status of healthy or low grade dysplastic epithelium. This effect can be addressed by a more substantial stain augmentation and a more extensive targeted training on dark-stained data, but requires retraining the network. An alternative would be to apply stain normalization during inference for which no retraining is required. An example of this is the use of Cycle-GANS as proposed by^41^. In future work we will train with more dark-stained data and try stain normalization. Other sources of errors are related to incidental artifacts such as small air bubbles in the WSI’s and partly with staining (artifacts) (as shown in Fig. 7d). Suboptimal performance due to these kind of artifacts can be countered by adopting some form of quality control of digital slides, or by explicitly introducing regions with artifacts in the training set.

The network system generates relatively the most errors in the groups healthy and hyperplasia epithelium and the least in the low grade dysplasia and high gade dysplasia/tumor classes, but misses on the latter class should be considered heavily. To the extent that those errors occur on the tumor class, for example in biopsies where low grade dysplasia and high-grade dysplasia both occur, they are rather related to the classifier’s limitations and the same applies to problems with the classification of hyperplasia. We did not include the latter class in the development of the segmentation algorithm (out of necessity, i.e., in connection with too little visual material), but we did include it in the classification task, which relies on the aforementioned features during execution. With this approach we were able to correctly classify 29 cases of the 36 hyperplasia cases (see Fig. 7b), but the system appears to be insufficiently able to distinguish this class from the low grade dysplasia class. It is therefore recommended that this tissue type—in addition to the other epithelial types—be included directly in the segmentation. Since information about low-grade dysplasia is only present in the normalized histogram, we expect that adding more features about these classes could improve the classification output. However, because our primary objective here is to develop a broad segmentation model, broadening of the model and optimization of the segmentation performance, including on the necrosis class, will be our first point of attention in the future.

## Conclusion

In this paper, we have compared performance of semantic segmentation models for histopathology images in CRC using four different loss functions, three of which are per-pixel categorical cross-entropy (related) and one metric sensitive loss (Lovasz-softmax loss). All networks were trained on a single center dataset and evaluated on three medical segmentation tasks from multiple centers. We found no major differences between the performance of the different loss models, but saw a consistently best performance of the Lovasz-softmax function on all tasks and a variable task-dependent prediction quality of the Bi-tempered loss. We definitely see the use of the Lovasz-softmax loss as the better alternative for both the categorical cross-entropy and the Focal loss, but also for the Bi-tempered loss, provided there is no significant degree of noise in the dataset. In practice the Lovasz-softmax performs equally or better than the other losses, and visually gives a more accurate, and cleaner segmentation result, and a better definitions of the borders. Since there are no hyperparameters to tune, it is easier to use in comparison to the Focal and Bi-tempered loss.

We used the model trained with the Lovasz-softmax loss to segment a series of more than thousand biopsies and classified them into four classes, in line with current pathology reports, using a simple classifier and a handful of features, derived directly from the segmentation output. We showed that with a good segmentation as base one can obtain very good results on a downstream task. The classifier could potentially support pathologists in diagnosing colon biopsies in the context of population screening.

## Appendix

See Table 3.

**Table 3:** Overview of all datasets with technical information.

Purpose	Center origin	# of slides	Tissue type	Annotation types	Scanner	Scanning resolution (μm/px)
Training $D_{seg}$	Radboud University Medical Center	40	Resections & biopsies	Tissue annotations	Pannoramic P250 Flash II (3D-Histech, Hungary)	0.24
Testing segmentation network $D^{test}_{seg}$	Radboud University Medical Center	12	Resections & biopsies	Tissue annotations	Pannoramic P250 Flash II (3D-Histech, Hungary)	0.24
	Eindhoven Medical center	4	Resections	Tissue annotations	Pannoramic P250 Flash II (3D-Histech, Hungary)	0.24
	Utrecht University Medical Center	5	Resections	Tissue annotations	Pannoramic P250 Flash II (3D-Histech, Hungary)	0.24
	Leiden University Medical Center	5	Resections	Tissue annotations	IntelliSite (Philips, the Netherlands)	0.24
	University of Bayreuth	3	Resections	Tissue annotations	NanoZoomer 2.0 HT (Hamamatsu, Japan)	0.24
Testing biopsy classifier $D_{cls}$	Cannizzaro Hospital	1054	Biopsies	Slide level label	Aperio AT2 (Leica Biosystems)	0.24

### Overview of proposed method

See Fig. 8.

**Figure 8:**
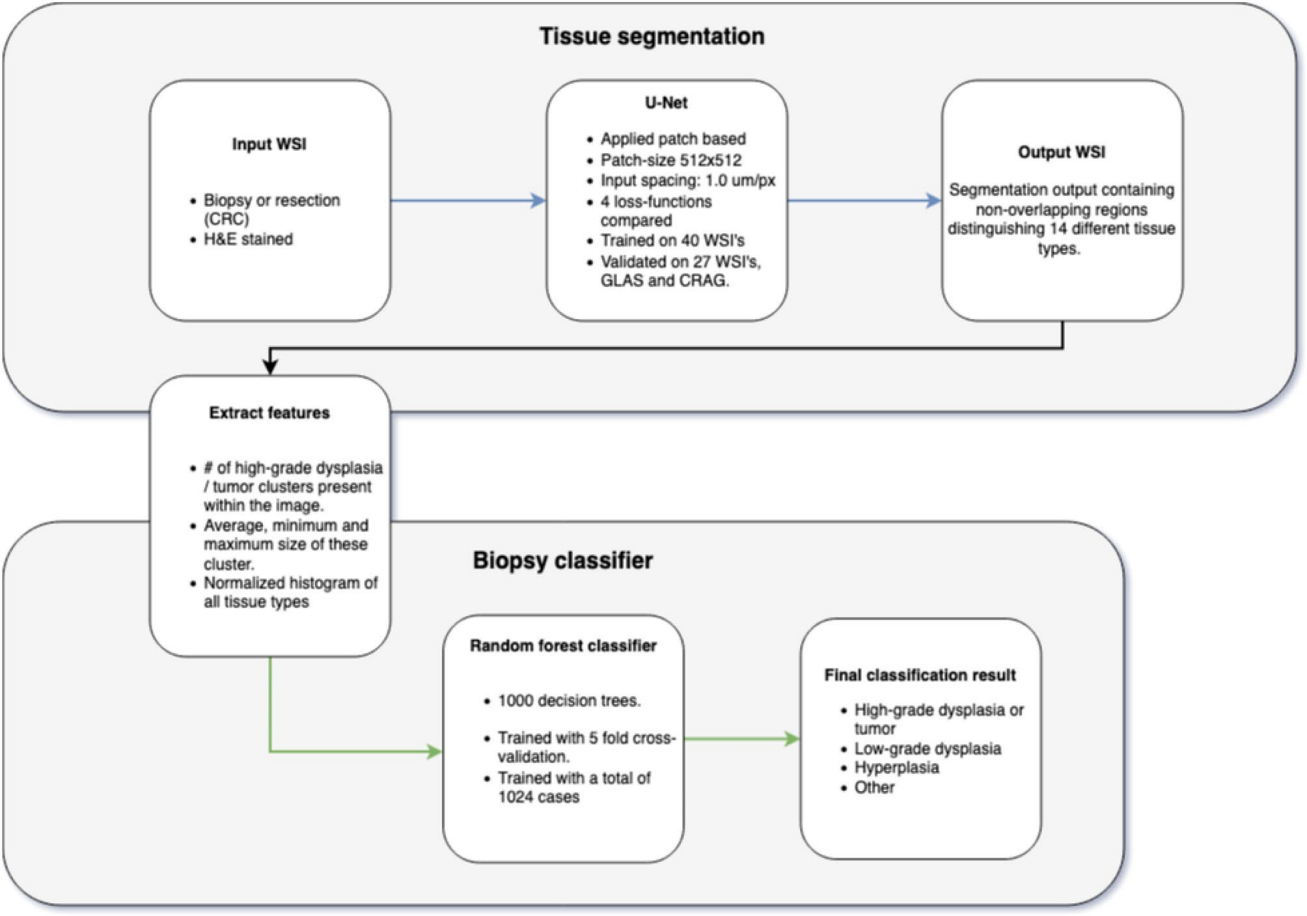
Blockdiagram of the entire pipeline. We start with the segmentation process, which segments 14 different tissue types. From the segmentation map a set of features is extracted: (**a**) the normalized histogram of all tissue types, (**b**) the number of high-grade dysplasia/tumor clusters, (**c**) the average, minimum, and maximum size of these clusters. These features are processed by a random forest classifier that gives the final classification. This figure has been created with DrawIO (https://www.draw.io/index.html).

### Overview of DICE-scores per center

See Fig. 9.

**Figure 9:**
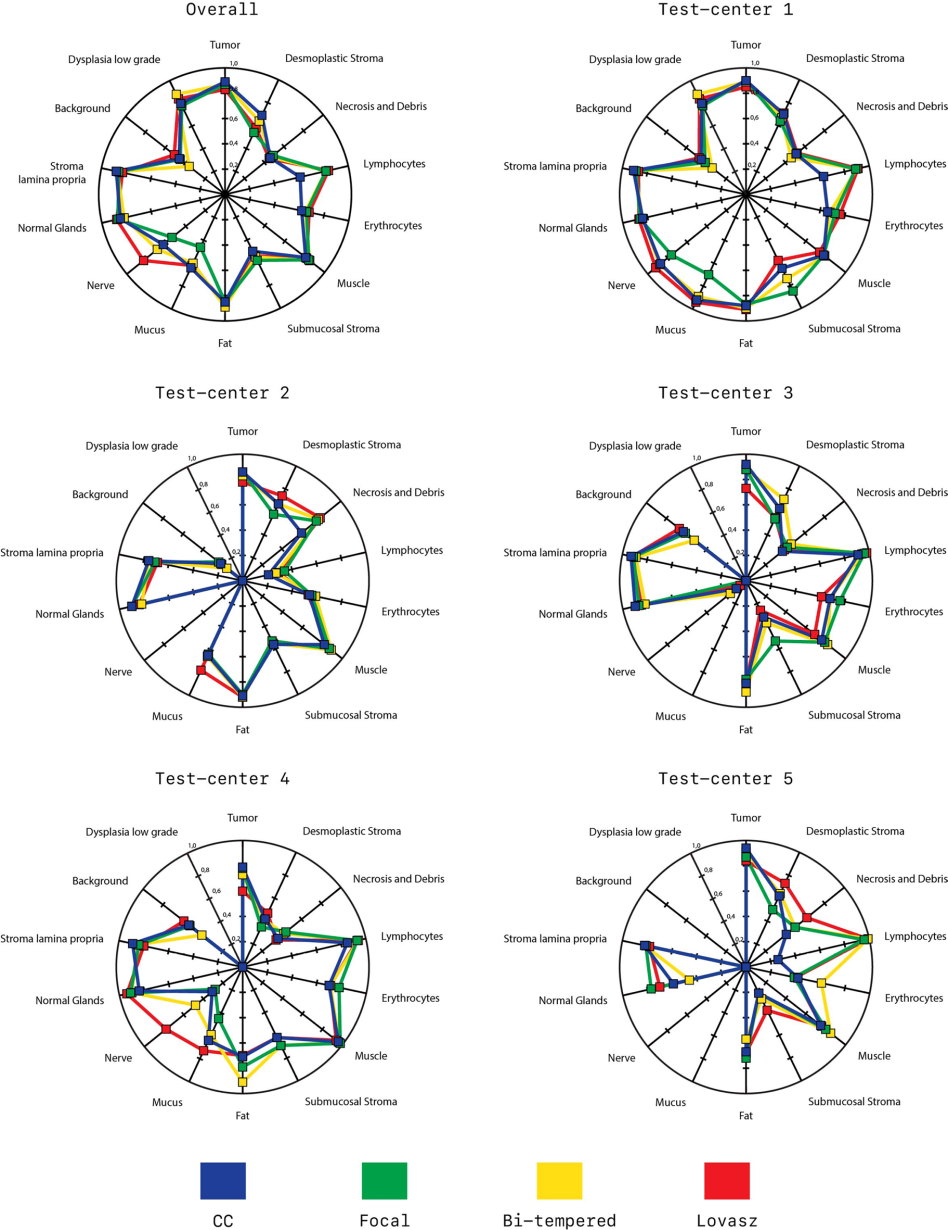
DICE scores of the different loss functions per class of; top-left) the entire test-set or per test-center in the private dataset. Note that if a specific class is not present the DICE-score is zero. This figure has been created with Adobe Illustrator 23 (https://www.adobe.com/products/illustrator.html).

## Data Availability

The GLAS and CRAG datasets are pubically available on https://warwick.ac.uk/fac/cross_fac/tia/data/glascontest/ and https://warwick.ac.uk/fac/sci/dcs/research/tia/data/mildnet. The datasets used to train the semgentation algorithm are available from the corresponding author on reasonable request.

## Code availability

The segmentation model is available for research use on https://grand-challenge.org/algorithms/colon-tissue-segmentation/.
